# Parental Post-traumatic Stress Disorder Symptoms Are Related to Successful Aging in Offspring of Holocaust Survivors

**DOI:** 10.3389/fpsyg.2017.01099

**Published:** 2017-06-29

**Authors:** Amit Shrira, Liat Ayalon, Moshe Bensimon, Ehud Bodner, Tova Rosenbloom, Gal Yadid

**Affiliations:** ^1^The Interdisciplinary Department of Social Sciences, Bar-Ilan UniversityRamat-Gan, Israel; ^2^School of Social Work, Bar-Ilan UniversityRamat-Gan, Israel; ^3^Department of Criminology, Bar-Ilan UniversityRamat-Gan, Israel; ^4^Department of Music, Bar-Ilan UniversityRamat-Gan, Israel; ^5^Department of Management, Bar-Ilan UniversityRamat-Gan, Israel; ^6^Leslie Susan Gonda (Goldschmied) Multidisciplinary Brain Research Center and The Mina & Everard Goodman Faculty of Life Sciences, Bar-Ilan UniversityRamat-Gan, Israel

**Keywords:** Holocaust, intergenerational transmission, parental PTSD, secondary traumatization, successful aging

## Abstract

A fascinating, yet underexplored, question is whether traumatic events experienced by previous generations affect the aging process of subsequent generations. This question is especially relevant for offspring of Holocaust survivors (OHS), who begin to face the aging process. Some preliminary findings point to greater physical dysfunction among middle-aged OHS, yet the mechanisms behind this dysfunction need further clarification. Therefore, the current studies assess aging OHS using the broad-scoped conceptualization of successful aging, while examining whether offspring successful aging relates to parental post-traumatic stress disorder (PTSD) symptoms and offspring’s secondary traumatization symptoms. In Study 1, 101 adult offspring (mean age = 62.31) completed measures of parental PTSD, secondary traumatization, as well as successful aging indices – objective (medical conditions, disability and somatic symptoms) and subjective (perceptions of one’s aging). Relative to comparisons and OHS who reported that none of their parents suffered from probable PTSD, OHS who reported that their parents suffered from probable PTSD had lower scores in objective and subjective measures of successful aging. Mediation analyses showed that higher level of secondary traumatization mediated the relationship between parental PTSD and less successful aging in the offspring. Study 2 included 154 dyads of parents (mean age = 81.86) and their adult offspring (mean age = 54.48). Parents reported PTSD symptoms and offspring reported secondary traumatization and completed measures of objective successful aging. Relative to comparisons, OHS whose parent had probable PTSD have aged less successfully. Once again, offspring secondary traumatization mediated the effect. The findings suggest that parental post-traumatic reactions assessed both by offspring (Study 1) and by parents themselves (Study 2) take part in shaping the aging of the subsequent generation via reactions of secondary traumatization in the offspring. The studies also provide initial evidence that these processes can transpire even when offspring do not have probable PTSD or when controlling offspring anxiety symptoms. Our findings allude to additional behavioral and epigenetic processes that are potentially involved in the effect of parental PTSD on offspring aging, and further imply the need to develop interdisciplinary interventions aiming at promoting successful aging among offspring of traumatized parents.

## Introduction

In view of the recent, extensive increase in life expectancy, there is a growing need to understand how people can preserve their health and age successfully. Exposure to massive traumatic events such as genocide can shape the aging process of survivors, but also affect the aging process of their adult offspring. Whereas there is evidence that individuals who were previously exposed to massive trauma suffer from greater physical (Keinan-Boker et al., 2009; Iecovich and Carmel, 2010) and psychological morbidity (Steel et al., 2009; Barel et al., 2010) in late life, little is known about whether and how indirect exposure to genocide affects the aging process of subsequent generations who were not directly exposed. Therefore, this research aims to advance our knowledge regarding if, when and how exposure to genocide affects the aging process of adult offspring of Holocaust survivors (OHS).

The extreme nature of the Holocaust, and the fact that an increasing number of OHS have begun to cope with the aging process, makes the study of this group pertinent to cardinal questions about the effects of trauma and its transmission on aging. First, the study aims to pinpoint OHS who are at higher risk of vulnerability in late-life, as well as those who manifest resilience. Second, the study aims to assess a major mechanism through which the effect of ancestral trauma might linger across the lifespan of generations to manifest itself in old age – secondary traumatization.

The literature on physical and psychological morbidity of middle-aged OHS generally shows conflicting evidence (for reviews, see Danieli, 1998; Solomon, 1998; Kellermann, 2009; Shmotkin et al., 2011). An extensive meta-analysis (van IJzendoorn et al., 2003), which looked at 32 samples published by 2003, failed to find any difference between OHS and comparisons across several measures of psychosocial functioning. That meta-analysis however, did find greater distress among OHS who coped with traumatic and stressful events relative to comparison offspring (Non-OHS), who coped with the same hardships. A recent literature review that included 18 studies on community-dwelling OHS (Lindert et al., 2016) concluded that half of the works found some sign of psychopathology among OHS, and half failed to find such signs. Moreover, studies rated as methodologically superior largely belonged to the latter half. The few studies that assessed physical health among adult OHS also showed mixed results. Increased physical morbidity was documented among middle-aged and older OHS, especially when mothers (Flory et al., 2011), or both parents (Shrira et al., 2011a), were exposed. However, another study failed to find significant differences in physical morbidity among OHS (Levav et al., 2007).

In view of the conflicting findings, scholars proposed that research efforts should be allocated from the general question of whether OHS are more vulnerable, to more specific questions, asking in which families should we expect to see trauma transmission, and what are the mechanisms by which transmission transpire (Kellermann, 2009; Danieli et al., 2016). It seems that intergenerational transmission of trauma should not be viewed as an inevitable consequence of parental exposure *per se*, but should rather be an outcome of an unresolved attempt by the parent to cope with the trauma. Accordingly, parental post-traumatic stress disorder (PTSD) has been generally associated with an increased risk for pathology in young offspring (Leen-Feldner et al., 2013; Lambert et al., 2014). OHS also showed increased vulnerability to PTSD and other psychiatric disorders, especially when they perceived both their parents to have PTSD (Yehuda et al., 2008), or perceived one of the parents to have a negative parental style (Danieli et al., 2016), meaning parents characterized as being stuck in the trauma, or those who are numb and emotionally detached. Still, as aforementioned, OHS, some with post-traumatic parents, begin to cope with their own aging process, while sometimes serving as caregivers to their elderly parents (Anderson et al., 2013; Samson et al., 2013), and questions remain regarding when and how ancestral trauma lingers to affect these offspring in late life.

We propose that OHS with post-traumatic parents should show less successful aging than OHS or comparisons (whose parents were not directly exposed to the Holocaust) without post-traumatic parents. We further propose that less successful aging would be evident even when offspring themselves do not suffer from PTSD, as secondary traumatization symptoms may be detrimental to the aging process, and will therefore mediate the relationship between parental PTSD and offspring less successful aging. Contrary, OHS whose parents do not suffer PTSD, and are therefore less prone to secondary traumatization, should age as successfully as comparisons. Such OHS may even be characterized by resilience extending from parents, who overcame the trauma. We next elaborate on our main mediator – secondary traumatization – and the main outcome – successful aging.

Secondary traumatization refers to symptoms of distress and behaviors that result from close or extended contact with a traumatized individual (Figley, 1995; Motta et al., 2001). With the exception that these symptoms are generated from knowledge of another person’s trauma and not from direct experience, they parallel those of PTSD. Surprisingly, little is known regarding secondary traumatization among OHS. In the few studies that did examine it, OHS had lower secondary traumatization when they reported a more free-flowing exchange of emotional and factual information between family members (Giladi and Bell, 2013), or when they remembered that their parents were careful not to flood them with their burden (Letzter-Pouw et al., 2014).

Whereas numerous studies connect PTSD to greater physical morbidity, premature mortality (Pacella et al., 2013; Solomon et al., 2014; Lohr et al., 2015), and negative evaluations of aging (Pietrzak et al., 2014; Avidor et al., 2016), secondary traumatization is rarely considered in the context of aging. Nevertheless, preliminary findings show that OHS, who reported their parents communicated their trauma in an intrusive way (cf. Wiseman et al., 2002), also reported higher secondary traumatization, which in turn related to increased physical morbidity and negative perceptions regarding aging (Shrira, 2016).

When assessing the effect of parental PTSD and secondary traumatization on offspring aging, we chose to take a broad-scope view on aging by focusing on successful aging. A well-received operationalization of successful aging was proposed in the groundbreaking work of Rowe and Kahn (1987). They maintained that disease, disability, and decline could be considered as usual, but not inevitable processes that occur in old age. Successful aging, on the other hand, was described as the combination between freedom from disease and disability and active engagement with life. However, critics pointed that this definition depicts an extraordinary or exceptional aging, rather than successful aging, as very few older people are able to maintain high levels of functioning to be labeled “successful” (Martin et al., 2015). Therefore, scholars recently suggested the need to move from a dichotomous operationalization of successful aging (i.e., either successful or unsuccessful aging) to a continuous one, as even when older adults experience limitations in one domain of functioning, they may perform relatively well in other domains (Kok et al., 2015). In addition, although successful aging as an objective construct dominated the field for years, more recently, some emphasized the need to incorporate the subjective dimension of older person’s own criteria and perceptions to successful aging (Pruchno et al., 2010). Among others, these perceptions may refer to the way one evaluates one’s own aging experience (Pruchno et al., 2010), and the extent one can focus on age-related benefits and the potential for late-life growth (Laidlaw et al., 2007). Following the above, we decided to operationalize successful aging as a continuous, multi-dimensional construct that incorporates both objective and subjective success (cf. Pruchno and Wilson-Genderson, 2015).

**FIGURE 1:**
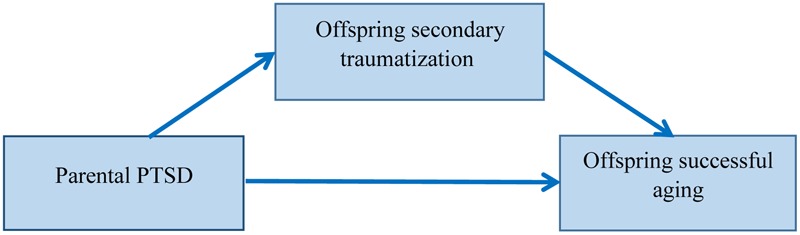
The research model, according to which parental PTSD relates to offspring successful aging via offspring secondary traumatization.

To recapitulate, we propose a model in which parental PTSD relates to offspring successful aging via secondary traumatization (see **Figure 1**). We therefore hypothesized that OHS with parental PTSD will present less successful aging relative to comparisons, whereas OHS without parental PTSD will age as successfully as comparisons. The effect of parental PTSD on offspring successful aging will be evident even when the offspring themselves do not suffer from probable PTSD, or after controlling for their anxiety symptoms. Second, we hypothesized that secondary traumatization symptoms will mediate the effect of parental PTSD on offspring successful aging. We examined our hypotheses in two separate studies. Study 1 assessed parental PTSD based on offspring reports, whereas Study 2 assessed parental PTSD directly by interviewing the parents themselves.

## Study 1

As noted above, in this study we asked offspring to report secondary traumatization symptoms and complete various indices of successful aging. We also asked offspring to report parental PTSD symptoms. Some scholars noted that offspring perceptions merit assessment in themselves, as such perceptions might better predict offspring’s behavior than parents’ actions (e.g., Kellermann, 2001). Indeed, offspring were previously asked to report parental behaviors (Letzter-Pouw et al., 2014), parental styles of coping with the trauma (Danieli et al., 2016) and parental PTSD symptoms (Yehuda et al., 2008). Yehuda et al. (2006) further validated offspring reports by demonstrating significant correlations between OHS reports regarding their parents’ PTSD symptoms with parents’ self-reports using the Clinician Administered PTSD Scale.

### Materials and Methods

#### Participants and Procedure

The study examined a convenience sample of 101 community-dwelling participants. Sixty-nine respondents were offspring with two Holocaust survivor parents, and 32 were comparisons, with two parents without a Holocaust background.

Inclusion criteria were being born after 1945, having parents who were alive during World War II (1939–1945), being a Jewish Israeli from families of European origin, living in Israel, and being a Hebrew-speaker. Exclusion criteria included reporting to have been diagnosed by a psychiatrist or a psychologist with major mental disorders including major depressive disorder, bipolar disorder, psychotic disorder or PTSD. Similar single-item screening is a quick means to screen for mental disorders and has been proven to be valid (e.g., Chochinov et al., 1997; Farvolden et al., 2003; Donker et al., 2009). In addition, exclusion criteria included scoring higher than 30 on the PTSD Checklist for DSM-5 (Weathers et al., 2013), as sum scores of 31 or higher indicate probable PTSD (cf. Blevins et al., 2015; Bovin et al., 2016).

Among OHS the mean age was 62.24 (*SD* = 5.28), 58.0% were women, 1.4% had a below high-school education, 8.7% completed high school, 89.9% had an above-high-school education, 88.4% were married, and 68.1% reported that they are currently employed or self-employed. Among comparisons the mean age was 62.46 (*SD* = 5.13), 62.5% were women, 6.3% had a below high-school education, 12.5% completed high school, 81.3% had an above-high-school education, 84.4% were married, and 81.3% reported that they are currently employed or self-employed. The groups did not differ significantly on age (*t*[99] = 0.19, *p* = 0.84), gender (χ^2^[1] = 0.18, *p* = 0.66), education (χ^2^[2] = 2.20, *p* = 0.33), marital status (χ^2^[3] = 2.18, *p* = 0.53), or employment status (χ^2^[1] = 1.88, *p* = 0.17).

Parental Holocaust background was determined by asking the offspring whether their parents were under Nazi or pro-Nazi occupation or domination during World War II. Fifty-two percent of OHS reported their parents were in concentration camp, 76.8% in work camp, 55.0% in ghetto, 39.1% in hiding, 11.5% with the partisans, 20.2% reported their parents used false papers, and 27.5% reported their parents were in permanent escape.

As we mainly searched for middle age or older individuals, participants were approached through networks of institutions related to aging (e.g., Israel Gerontological Data Center, The Herczeg Institute on Aging), and were invited to take part in a study assessing coping with difficult life events. Eligibility criteria were detailed. From January until March 2016, eligible participants entered a link to an online informed consent form, which noted that the questionnaire includes mentally difficult queries. The informed consent form also included the contact details of the principal investigator (A.S.) and of several organizations providing mental health services. Participants could approach any of the above in case of feeling distressed by the questionnaire. After noting their agreement to participate, participants completed an online questionnaire. Participants’ confidentiality was guaranteed, as their names were not noted on the questionnaire. In case participants voluntarily agreed to be included in future studies, they supplied their contact details following the completion of the questionnaire. The study received ethical approval by an ethic review committee at Bar-Ilan University.

#### Measures

Parental PTSD symptoms were separately completed for each of the parents using the PTSD Checklist for DSM-5 (Weathers et al., 2013). OHS were asked to refer to parental symptoms due to the Holocaust, and comparisons were asked to refer to parental symptoms due to the most traumatic event the parent underwent. Comparisons were further asked to note the traumatic event experienced by the parent that they chose to refer to. When looking at the events reported for both parents, close to a third of the comparisons (29.7%) referred to the sudden loss of a loved one, 18.8% referred to exposure to warfare, 12.5% to life threatening illness, 3.1% to serious accident, 3.1% to a life threatening event that occurred to a loved one, 1.6% to physical assault, and 9.4% referred to other life threatening events. Respondents indicated how much they perceived each of their parents to had been bothered by each of the 20 DSM-5 (American Psychiatric Association [APA], 2013) PTSD symptoms using a 5-point scale ranging from 0 (*not at all*) to 4 (*extremely*). Scores were based on the sum of ratings. Probable parental PTSD was determined as present for scores of 31 or higher (cf. Blevins et al., 2015; Bovin et al., 2016). Cronbach’s α for maternal and paternal ratings was 0.94 and 0.93, respectively. Previous studies have used the Hebrew version of the PTSD Checklist for DSM-5 (e.g., Shrira et al., 2016).

Secondary traumatization was assessed by the modified Secondary Trauma Questionnaire (Motta et al., 2001). Participants rated the frequency in which they experienced 18 symptoms of distress, due to traumatic events experienced by one of their parents, using a scale ranging from 1 (*never or rarely*) to 5 (*very often*). Following Giladi and Bell (2013), OHS were asked to relate specifically to the Holocaust as the traumatic event, and participants of the comparison group were instructed to refer to the most traumatic event the parent underwent. Participants were asked to refer to the parent to whom they felt the closest. About half (55.9%) chose to refer to their mother and the others (44.1%) referred to their father. There was no significant difference in the choice of parent between OHS and comparisons (χ^2^[1] = 0.21, *p* = 0.64). The final score was based on the sum of answers. Higher scores reflect higher secondary traumatization. Cronbach’s α was 0.84. Previous studies have used the Hebrew version of this measure (e.g., Dekel et al., 2007; Shrira, 2016).

Following Pruchno and Wilson-Genderson (2015), we assessed objective successful aging (objective success) according to medical conditions, disability and somatic symptoms. These are three constituents, which are not subject to value judgments, can be collected through reliable self-report measures, and are varied in advanced age. The measure of medical conditions was computed by summing the number of 14 medical conditions participants reported to have been diagnosed by a physician (Shrira et al., 2011b). These conditions included heart disease, high blood pressure, high cholesterol, stroke or cerebral vascular disease, diabetes or high blood sugar, chronic lung disease such as chronic bronchitis or emphysema, asthma, arthritis, osteoporosis, cancer, or malignant tumor, stomach or duodenal ulcer or peptic ulcer, Parkinson disease, cataracts, and hip fracture or femoral fracture.

The measure of disability (functional limitations) adapted from Nagi (1976) is the mean of limitations in performing five physical activities, rated on a scale ranging from 1 (*not at all*) to 5 (*to a very large extent*). These activities include stooping, kneeling, or crouching, reaching or extending arms above shoulder level, pulling or pushing heavy objects, lifting or carrying heavy weights, and picking up a small coin from a table (Cronbach’s α was 0.76). Previous studies have used the Hebrew version of this measure (e.g., Shrira, 2016).

The measure of somatic symptoms was based on the somatization subscale derived from the 18-item Brief Symptom Inventory (BSI-18; Derogatis, 2001). We computed the mean of six items rated on a scale ranging from 0 (*not at all*) to 4 (*very much*) with a Cronbach’s α of 0.69. Previous studies have used the Hebrew version of this measure (e.g., Bodner et al., 2015).

In order to compute the overall objective successful aging score, we first standardized the three scores (medical conditions, disability and somatic symptoms), and then averaged the standardized scores. We then multiplied the averaged standardized score by -1, so that high scores will reflect greater objective success.

Subjective successful aging (subjective success) was assessed with the Attitudes to Aging Questionnaire (AAQ; Laidlaw et al., 2007). This is a 24-item questionnaire that includes three subscales (eight-items in each subscale): psychological loss, psychological growth and physical change. Psychological loss refers to primarily seeing old age as a negative experience involving psychological and social losses (“Old age is a depressing time of life”). Psychological growth refers to positive gains in relation to self and to others that may have been a surprise about aging (“There are many pleasant things about growing older”). Physical change refers to being focused on health, exercise and the experience of aging (“Growing old has been easier than I thought”). We computed the mean items in each scale rated on a scale ranging from 1 (*completely disagree*) to 5 (*completely agree*). As correlations showed that psychological loss is relatively separate from the other two subscales, we opted not to average the three subscale scores together. The psychological loss score was computed with reverse-coded items, so higher score reflected higher subjective success as the other two scores. The Cronbach’s α was 0.86, 0.82, and 0.90 for psychological loss, psychological growth and physical change, respectively.

#### Data Analysis

We first divided our respondents according to probable parental PTSD: OHS who reported that at least one parent had probable PTSD (*n* = 24), OHS who reported that none of their parents had probable PTSD (*n* = 45), and comparisons (none of them reported that any of their parents had probable PTSD; *n* = 32). In the main analyses, we compared these groups. Nevertheless, we also performed supplementary analyses in which we separated OHS with paternal only (*n* = 7), maternal only (*n* = 12) and both paternal and maternal PTSD (*n* = 5). It should be noted that the findings from these supplementary analyses should be viewed with caution due to the small groups’ size.

To test the first hypothesis, we performed a multivariate analysis of variance (MANOVA) with *post hoc* Bonferroni-corrected pairwise comparisons, examining differences in successful aging and secondary traumatization among OHS with parental PTSD, OHS without parental PTSD and comparisons. To test the second hypothesis, PROCESS (Hayes, 2013) was used for mediational analyses in which groups were the independent, secondary traumatization was the mediator, and successful aging indices were the dependents. PROCESS examines mediation through an indirect effect analysis using a bias-corrected bootstrap with 5,000 resamples. Through the application of bootstrapped confidence intervals, it is possible to avoid power problems introduced by asymmetric and other non-normal sampling distributions of an indirect effect (MacKinnon et al., 2004).

Using G^∗^Power 3.1.9.2 (Faul et al., 2009), we performed a *post hoc* power analysis for a MANOVA with five dependents, three groups, 101 participants, set to an effect size of η^2^ = 0.06 (i.e., medium). This effect size was based on those found in previous studies that are similar to the current study (e.g., Shrira, 2016). This analysis yielded power of 0.997. Therefore, our sample was sufficient in order to detect such effects.

### Results and Discussion

The three groups did not significantly differ in age (*F*[2,98] = 2.04, *p* = 0.13), gender (χ^2^[2] = 0.18, *p* = 0.91), education (χ^2^[4] = 3.14, *p* = 0.53), marital status (χ^2^[6] = 8.37, *p* = 0.21), or employment status (χ^2^[2] = 2.46, *p* = 0.29). **Table 1** presents the results of the MANOVA comparing the groups on successful aging and secondary traumatization.

**Table 1:** Results of multivariate analysis of variance comparing groups on successful aging and secondary traumatization: Study 1.

Variable	OHS with parental PTSD *M* (*SD*)	OHS without parental PTSD *M* (*SD*)	Comparisons without parental PTSD *M* (*SD*)	*F*	*p*	η^2^
Objective success	-0.47 (0.89)^a^	0.20 (0.55)^b^	0.02 (0.59)^b^	8.08	0.001	0.151
Psychological loss (reverse coded)	3.93 (0.76)^a^	4.50 (0.55)^b^	4.28 (0.65)^a,b^	6.08	0.003	0.118
Psychological growth	3.55 (0.97)	3.65 (0.86)	3.62 (0.95)	0.08	0.919	0.002
Physical change	3.35 (0.87)	3.59 (0.94)	3.45 (0.92)	0.55	0.576	0.012
Secondary traumatization	31.04 (9.04)^a^	22.11 (4.46)^b^	20.53 (2.95)^b^	25.50	<0.0001	0.359

*n is 24, 44, and 26 for OHS with parental PTSD, OHS without parental PTSD and comparisons, respectively. Wilks’ λ (2,91) = 0.57, *p <* 0.0001; Means that do not share letters significantly differ from each other in a *post hoc* Bonferroni test.*

The groups significantly differed in objective success, psychological loss and secondary traumatization. Bonferroni *post hoc* tests showed that OHS with parental PTSD had lower objective success and higher secondary traumatization than the other two groups. OHS with parental PTSD also reported lower subjective success in the reverse-coded psychological loss subscale compared with OHS without parental PTSD. Subsequent mediation analyses were performed with objective success and psychological loss.

The effect of group on objective success became non-significant after including secondary traumatization (*B* = -0.09, *t* = -0.82, *p* = 0.41). Similarly, the effect of group on psychological and social loss became non-significant after including secondary traumatization (*B* = -0.01, *t* = -0.16, *p* = 0.87). Secondary traumatization significantly mediated the relationship between groups and objective success (indirect effect = -0.15, 95%LLCI = -0.33, 95%ULCI = -0.03), as well as the relationship between group and psychological and social loss (indirect effect = -0.15, 95%LLCI = -0.32, 95%ULCI = -0.03). The mediation effect of secondary traumatization on objective success and on psychological loss resulted in effect size of κ^2^ (Preacher and Kelley, 2011) equal to 0.13 and 0.14, respectively. Thus, according to Preacher and Kelley’s (2011) suggestions, the size of both mediation effects of secondary traumatization may be labeled as lying in the medium range.

Supplementary analysis separately accounting for paternal and maternal PTSD (with five groups: OHS with both paternal and maternal PTSD, OHS with paternal PTSD, OHS with maternal PTSD, OHS without parental PTSD, and comparisons without parental PTSD) found significant group effects for objective success (*F*[4,89] = 4.58, *p* = 0.002, η^2^ = 0.171), psychological and social loss (*F*[4,89] = 2.98, *p* = 0.023, η^2^ = 0.118) and secondary traumatization (*F*[4,89] = 15.65, *p* < 0.0001, η^2^ = 0.413). No other significant differences were found. Bonferroni *post hoc* tests showed that OHS with maternal PTSD had lower objective success than comparisons and OHS with no parental PTSD. Moreover, OHS with maternal PTSD and OHS with two parents with probable PTSD reported higher secondary traumatization than both comparisons and OHS with no parental PTSD. There were no significant *post hoc* group differences in psychological and social loss.

The findings of Study 1 supported both our hypotheses. First, compared to OHS without paternal PTSD and comparisons, OHS who reported that at least one of their parents suffered from probable PTSD showed lower objective success. Relative to their counterparts without parental PTSD, OHS with parental PTSD were also more inclined to perceive old age as a negative experience involving psychological and social loss. Second, secondary traumatization mediated these group effects. Next, in Study 2 we aimed to replicate some of these findings in a sample composed of dyads of parents and offspring.

## Study 2

Although transmission of trauma is best captured when parents and offspring are assessed in tandem, so that the status of one generation can be correlated with that of the other, most works focused on one generation, either Holocaust Survivors (HS) or OHS. There have been few multi-generational studies in this specific population (Fridman et al., 2011; Letzter-Pouw and Werner, 2013; Yehuda et al., 2016), and none of them, to the best of our knowledge, has focused on the relationship between parental PTSD and offspring successful aging. In this study, we assessed PTSD symptoms in parents, and further assessed secondary traumatization and successful aging in offspring. Therefore, information is not solely obtained from a single informant (as in Study 1), but obtained from both parents and offspring.

### Materials and Methods

#### Participants and Procedure

The study examined a convenience sample of 308 community-dwelling participants, who consisted 154 dyads of parents and adult offspring. Ninety dyads included HS and OHS, and 64 dyads included comparison parents without a Holocaust background and their offspring.

Inclusion criteria for parents were being born before 1945, being a Jewish Israeli of European origin, living in Israel, and being a Hebrew-speaker. Inclusion criteria for offspring included being born after 1945, having two parents who were alive during World War II, being a Jewish Israeli from families of European origin, living in Israel, and being a Hebrew-speaker. There were no exclusion criteria, yet we accounted and controlled for anxiety symptoms in the main analyses (see below).

Eleven percent of parent-offspring dyads were father-son dyads, 22.1% were father-daughter dyads, 22.1% were mother-son dyads, and 44.8% were mother-daughter dyads. The ratio of the HS-OHS dyad types did not significantly differ from the comparison dyad types, χ^2^(3) = 1.84, *p* = 0.604.

**Table 2** presents the background characteristics of the study groups. HS were significantly older than comparison parents were, but the groups did not differ in gender, education, marital status, or employment status. The offspring groups did not differ in any of the background characteristics.

**Table 2:** Background characteristics of the study groups: Study 2.

	Survivor families		Comparison families			
	Parents	Offspring	Parents	Offspring	Comparison tests	
*N*	90	90	64	64	Parents	Offspring
Age					*t*(152) = -3.01, *p* = 0.003	*t*(152) = -1.51, *p* = 0.132
*M*	83.14	55.06	80.06	53.67		
*SD*	5.85	5.62	6.77	5.65		
Gender (%)					χ^2^(1) = 0.005, *p* = 0.946	χ^2^(1) = 1.23, *p* = 0.267
Woman	66.7	63.3	67.2	71.9		
Man	33.3	36.7	32.8	28.1		
Education (%)					χ^2^(2) = 3.94, *p* = 0.139	χ^2^(2) = 2.13, *p* = 0.344
Below high-school	50.6	2.3	38.1	6.3		
Full high-school	19.1	14.8	15.9	18.8		
Above high-school	30.3	83.0	46.0	75.0		
Marital status (%)					χ^2^(3) = 6.09, *p* = 0.107	χ^2^(4) = 4.50, *p* = 0.342
Married	37.1	88.9	53.1	87.5		
Widowed	58.4	3.3	43.8	3.1		
Divorced	4.5	6.7	1.6	3.1		
Single	0.0	1.1	1.6	3.1		
Partner	0.0	0.0	0.0	3.1		
Employment status (%)					χ^2^(1) = 2.32, *p* = 0.127	χ^2^(1) = 0.14, *p* = 0.708
Employed	6.7	86.7	14.1	88.7		
Not employed	93.3	13.3	85.9	11.3		

*N = 308.*

Holocaust background was determined by the parents’ presence under Nazi or pro-Nazi occupation or domination during World War II. Twenty two percent of HS reported being in concentration camp, 24.4% in work camp, 26.7% in ghetto, 38.9% in hiding, 3.3% with the partisans, 11.1% reported using false papers, 5.6% had permits to remain at their living place with restrictions, and 25.6% reported they were in permanent escape.

Undergraduate student research assistants were instructed to recruit eligible participants available in their surroundings (e.g., neighborhoods and large workplaces). The research assistants were instructed how to approach the interviewees and respond to potential difficulties. From January until April 2015, research assistants requested participants to take part in a study, which aims to examine how families cope with difficult life events. Participants read and signed an informed consent form, which also noted that the questionnaire includes queries regarding aging, death, various difficult life events and the Holocaust. Following that, participants entered through a link to an online questionnaire. The research assistants interviewed participants who could not complete the online questionnaire themselves. The participants’ confidentiality was guaranteed, as their names were not noted on the questionnaire. In the comparison group, parents participated before offspring, as the latter had to refer to a traumatic event that was reported by the former (in the Holocaust dyads, offspring were instructed to refer to the Holocaust). The study received ethical approval by an ethic review committee in Bar-Ilan University.

#### Measures

Parents completed the PTSD Checklist for DSM-5 (Weathers et al., 2013). In rating their symptoms, HS were asked to refer to the Holocaust, whereas comparison parents were asked to refer to the most traumatic event they underwent, which is also known to their offspring. When reporting their most traumatic event, close to half of the comparison parents (47.8%) referred to the sudden loss of a loved one, 13.0% referred to a serious accident, 10.9% referred to a life threatening event that occurred to a loved one, 8.7% referred to exposure to warfare, 6.5% to life threatening illness, 2.2% to physical assault, 2.2% to physical or sexual abuse, and 8.7% referred to other life threatening events. As in Study 1, probable PTSD was determined as present for scores of 31 or higher. Cronbach’s α was 0.91.

Offspring completed the Secondary Trauma Questionnaire (Motta et al., 2001). Participants were asked to specifically refer to the parent who participated in the study. OHS were asked to relate to the Holocaust as the traumatic event, and comparisons were instructed to refer to the traumatic event reported by their parents (the research assistants informed them of that event before they began to complete the questionnaire). Cronbach’s α was 0.89.

Offspring further completed the objective success measures as in Study 1. Cronbach’s α was 0.69 and 0.61 for disability and somatic symptoms, respectively. The final score of objective successful aging was computed as in Study 1. In contrast to Study 1, we did not assess PTSD symptoms in the offspring, yet offspring reported anxiety symptoms using the relevant subscale from the BSI-18 (Derogatis, 2001). We computed the mean of six items rated on a scale ranging from 0 (*not at all*) to 4 (*very much*) with a Cronbach’s α of 0.80.

#### Data Analysis

We divided our dyads according to probable parental PTSD: Holocaust dyads with a parent suffering from probable PTSD (*n* = 15), Holocaust dyads with a parent without probable PTSD (*n* = 75), and comparison dyads (*n* = 64; all of them with parents without probable PTSD).

To test the first hypothesis (the relationship between parental PTSD and offspring successful aging), we performed a MANOVA with *post hoc* Bonferroni-corrected pairwise comparisons, examining differences in successful aging and secondary traumatization among OHS with parental PTSD, OHS without parental PTSD and comparisons. To test the second hypothesis (the mediation effect of secondary traumatization), PROCESS (Hayes, 2013) was used for the mediational analysis in which groups were the independent variable, secondary traumatization was the mediator variable, and successful aging was the dependent variable. We further examined the above effects controlling for offspring’s anxiety symptoms.

Using G^∗^Power 3.1.9.2 (Faul et al., 2009), we performed a *post hoc* power analysis for a MANOVA with two dependent variables, three groups, 154 dyads, set to an effect size of η^2^ = 0.06. This analysis yielded power of 0.999. Therefore, our sample was sufficient in order to detect such effects.

### Results and Discussion

The three groups significantly differed in parental age (*F*[2,151] = 6.54, *p* = 0.002). Parents with probable PTSD were older than those in the comparison group. The groups did not significantly differ in parental gender (χ^2^[2] = 3.25, *p* = 0.19), education (χ^2^[4] = 4.70, *p* = 0.31), marital status (χ^2^[6] = 7.50, *p* = 0.27), or employment status (χ^2^[2] = 3.23, *p* = 0.19). The groups showed a marginally significant difference in offspring age (*F*[2,151] = 3.01, *p* = 0.052). The groups did not differ in offspring gender (χ^2^[2] = 2.04, *p* = 0.36), education (χ^2^[4] = 2.42, *p* = 0.65), marital status (χ^2^[8] = 11.71, *p* = 0.16) or employment status (χ^2^[2] = 0.14, *p* = 0.93). As parental age significantly differ between the groups, we also controlled for it alongside offspring’s anxiety.

**Table 3** presents the results of the MANOVA. The groups significantly differed in objective success and in secondary traumatization. Bonferroni *post hoc* tests showed that both OHS with parental PTSD and OHS without parental PTSD had lower objective success than comparison. The two OHS groups did not significantly differ from each other in objective success. OHS with parental PTSD had higher secondary traumatization than comparison. OHS without parental PTSD did not significantly differ from the other groups in secondary traumatization. After controlling for offspring’s anxiety and parental age, the group difference remained significant both in objective success (*F*[2,149] = 3.50, *p* = 0.033, η^2^ = 0.045) and secondary traumatization (*F*[2,149] = 5.13, *p* = 0.007, η^2^ = 0.064).

**Table 3:** Results of multivariate analysis of variance comparing groups on successful aging and secondary traumatization: Study 2.

Variable	OHS with parental PTSD *M* (*SD*)	OHS without parental PTSD *M* (*SD*)	Comparisons without parental PTSD *M* (*SD*)	*F*	*p*	η^2^
Objective success	-0.26 (0.95)^a^	-0.19 (0.79)^a^	0.28 (0.45)^b^	9.33	<0.0001	0.110
Secondary traumatization	32.20 (10.89)^a^	27.20 (9.81)^a,b^	24.20 (7.22)^b^	5.38	0.006	0.067

*N is 15, 75, and 64 for OHS with parental PTSD, OHS without parental PTSD and comparisons, respectively. Wilks’ λ (2,151) = 0.86, *p* < 0.0001; Means that do not share letters significantly differ from each other in a *post hoc* Bonferroni test.*

The effect of group on objective success remained significant after including secondary traumatization (*B* = -0.21, *t* = -2.70, *p* = 0.007), yet it decreased in its size relative to its effect before the inclusion of secondary traumatization (*B* = -0.34, *t* = -4.00, *p* = 0.0001). Secondary traumatization significantly mediated the relationship between groups and objective success (indirect effect = -0.13, 95%LLCI = -0.26, 95%ULCI = -0.04); κ^2^ (Preacher and Kelley, 2011) was 0.12, which could be labeled as lying in the medium range. The mediation effect remained significant after controlling for offspring’s anxiety and parental age (indirect effect = -0.04, 95%LLCI = -0.11, 95%ULCI = -0.01).

We could not compare the effect of maternal vs. paternal PTSD, as only two OHS had paternal PTSD. When performing the main analyses comparing OHS with maternal PTSD only (*n* = 13) to the other groups, results remained similar (results may be obtained from the authors upon request).

The findings of Study 2 largely replicated those of Study 1, but this time using parents’ actual reports of PTSD. Both OHS with and without parental PTSD had lower objective successful aging relative to comparisons. Although the two OHS groups did not significantly differ from each other, the former had the lowest objective success score. Again, secondary traumatization mediated the group difference. These findings remained significant after controlling for OHS’s anxiety symptoms. We now move to discuss the findings from both studies in more detail.

## General Discussion

To the best of our knowledge, the current studies are the first to substantiate the relationship between parental PTSD and the successful aging of OHS. Previous studies rarely assessed physical health among OHS (Levav et al., 2007; Flory et al., 2011; Fridman et al., 2011; Shrira et al., 2011a), and none of them looked at parental PTSD. In one study, lower health ratings were found among offspring with Holocaust survivor mothers (Flory et al., 2011), yet the results came from a select sample that might have been biased. Nevertheless, in a non-select, random sample, Shrira et al. (2011a) found that OHS, and especially those with two survivor parents, reported more medical conditions, greater use of medication and more physical symptoms than comparisons. Two additional studies that used non-select samples failed to find greater physical morbidity among OHS, although it should be noted that they included relatively younger samples (Levav et al., 2007; Fridman et al., 2011).

The current findings help to resolve the abovementioned contradictions, as it seems that offspring aging is not related to exposure *per se*, but to the parental reaction to the traumatic exposure, as manifested in parental PTSD symptoms. Thus, OHS with parental PTSD are at greater risk to show lower successful aging, whereas the aging of OHS without parental PTSD is generally more akin to that of comparisons. These findings complement to previous ones, pointing to lower successful aging among OHS who report that their parents flooded them with fragmented traumatic stories, mainly in a way that increased guilt and detachment from the parent (Shrira, 2016).

The current studies also show that the effects of parental PTSD remained significant even among offspring who do not suffer from probable PTSD (Study 1), or after controlling for offspring’s anxiety symptoms (Study 2). On the other hand, both studies showed that offspring’s secondary traumatization, which includes symptoms directly related to the parental trauma, significantly mediated the effect of parental PTSD. These findings add to those of a previous study, where secondary traumatization was found to mediate the effect of intrusive parental trauma-related communication on offspring’s successful aging (Shrira, 2016). Nevertheless, in that study, parental communication was reported by the offspring themselves. Here, we see that secondary traumatization serves as a central mediator both when parental PTSD is indirectly assessed based on offspring’s reports and when parental PTSD is directly assessed based on parents’ own reports.

Our main findings can be explained in several ways. For example, much like PTSD (Rosenbaum et al., 2015), chronic secondary traumatization can relate to maladaptive coping mechanisms, which ultimately serve as risk factors for metabolic disease, such as intake of high-fat food, and reduced physical activity levels. In addition, traumatized parents might have presented their offspring with a model of less successful aging; thus, shaping negative perceptions of aging and increase anxieties regarding physical weakness and death among offspring. When these offspring enter midlife and old age themselves, they find it harder to reconcile with losses, or to cope in ways that preserve functioning or promote growth. Some offspring, taught to distrust the world around them, may be reluctant to seek assistance, either as patients or as caregivers (Anderson et al., 2013; Samson et al., 2013). The negative perceptions of aging, which offspring hold, can accelerate greater physical health decline, as such perceptions are known to decrease control beliefs and hinder health behaviors (Westerhof and Wurm, 2015).

In a similar biological process to that which is characteristic to PTSD (Williamson et al., 2015), ongoing secondary traumatization symptoms throughout childhood and adulthood may relate to unmodulated levels of glucocorticoids and catecholamines in the hypothalamic-pituitary-adrenal-axis (HPA axis) and the autonomic nervous system, resulting in the dysregulation of blood pressure, heart rate variability, and insulin, glucose and lipids metabolism. When operated in a repeated cycles of stress, these effects cause an ‘allostatic load,’ or the wear and tear on the body systems leading to hypertension, hyperlipidemia, and atherosclerosis (McEwen, 1998) – conditions which are then translated to a less successful pattern of aging.

Recent findings suggest that epigenetic processes can help explain the connection between parental PTSD, secondary traumatization and biological dysregulation in offspring. Epigenetic processes refer to environmentally sensitive change that can alter gene expression and even includes genome reprogramming by processes such as DNA methylation, among others. There is compelling evidence that epigenetic processes may be involved in the passage of induced traits between generations. Although epigenetic processes are sensitive to environmental perturbation, they generate long-lasting signatures despite a shift in the environment originally responsible for initiating alteration (Szyf, 2015). Thereby, parents’ biological changes resulting from stress or psychopathology may impact parents’ gametes, the gestational uterine environment, or early postnatal care, and can thereby alter offspring biology (Bowers and Yehuda, 2016). Yehuda et al. (2014) provided the first evidence for the linkage between parental PTSD and epigenetic change in offspring. They found lower levels of DNA methylation (resulting in higher level of RNA expression) in the NR3C1 gene among OHS, who reported that both their parents suffered from PTSD. The lower DNA methylation (and higher RNA expression) of NR3C1 gene contributes to HPA-axis dysfunction. It is plausible to assume that the abovementioned, as well as other epigenetic markers (cf. Kellermann, 2013), generate biological dysregulation, which further induce phenotypic change by secondary traumatization, ultimately leading to less successful aging. Indeed, transmission of trauma can express across the many sites and trajectories of the human genome, as for example indicated by change in gene methylation in 1000s of sites examined in offspring of mothers exposed to the 1998 Quebec ice storm (Cao-Lei et al., 2015). These potential pathways to successful aging are yet to be examined. In addition to epigenetic processes and the long-term effects of HPA-axis dysregulation, future studies should also account for other possible pathways connecting parental PTSD at very early stages of the offspring’s life and offspring’s late-life physical morbidity. These include the effects of maternal stress during pregnancy on impaired uterine blood flow, low birth weight and pre-term birth (cf. Barker, 2004; Hazani and Shasha, 2008; Keinan-Boker, 2014).

The findings of the current studies should be viewed in light of their limitations. First, we used convenience internet samples, which were probably biased toward high-educated persons and high socioeconomic status. However, in contrast to many previous studies, the current studies did not specifically select participants from organizations related to the Holocaust, and the studies were presented to participants as general investigations relating to difficult or traumatic life events. This is of high importance, as studies that target participants from Holocaust-related organizations or gatherings are biased to produce larger effects of the Holocaust (Shmotkin and Lomranz, 1998) and its intergenerational transmission (van IJzendoorn et al., 2003). Second, the samples were not large enough to enable clear comparisons of parental and maternal PTSD. Previous studies show that maternal exposure (Flory et al., 2011) or maternal PTSD (Yehuda et al., 2008) may have stronger effects on offspring, whereas others found paternal effects as well (Letzter-Pouw et al., 2014). This issue should be further investigated in the context of offspring successful aging with larger samples. Third, parental PTSD and successful aging were based on self-reports. Although these variables are frequently assessed through self-report, psychiatric assessment of parental PTSD and biological indices of offspring physical health can further substantiate the link between the two. Fourth, although we verified that none of the offspring suffers from probable PTSD (Study 1), or controlled for general anxiety symptoms (Study 2), future studies should perform a more detailed screening of major mental disorders among offspring.

Aside from its limitations, the current research links parental PTSD and offspring successful aging. The studies also point to secondary traumatization as a major mechanism through which trauma transmits to affect the aging of offspring. In addition, the findings were replicated when using offspring reports and parents own reports of parental PTSD. Finally, the studies show that parental PTSD relates to offspring aging even when offspring do not have probable PTSD, or when offspring anxiety symptoms are controlled for. In view of the current findings, future studies need to assess the link between parental PTSD and offspring successful aging in other groups, in which ancestors were exposed to genocide and massive traumatic events, such as the Armenian genocide (Mangassarian, 2016), and the atrocities that occurred in Cambodia (Sack et al., 1995), Rwanda (Perroud et al., 2014), and former Yugoslavia (Schick et al., 2013). The effect of ancestral genocide on offspring aging might prove to be a worldwide phenomenon.

The current findings suggest the need to take an interdisciplinary approach in both preventive and reactive interventions aimed to increase successful aging of OHS. For example, preventive programs can include the dissemination of a campaign referring to specific groups of OHS, who may be at a high risk for late-life morbidity. Such a campaign may emphasize the need to approach psychosocial interventions, or perform preventive activities (e.g., initiating a healthy lifestyle, performing common screening tests; Keinan-Boker, 2014). In addition, reactive interventions may incorporate issues of successful aging and refer to perceptions of aging in light of findings showing that favorable perceptions have a beneficial impact on future functioning (Westerhof and Wurm, 2015).

## Ethics Statement

This study was carried out in accordance with the recommendations of the ethic review committee at Bar-Ilan University with written informed consent from all subjects. All subjects gave written informed consent in accordance with the Declaration of Helsinki. The protocol was approved by the ethic review committee at Bar-Ilan University.

## Author Contributions

All authors took part in planning the theoretical and conceptual basis for the study. AS performed the statistical analyses and wrote the first draft of the paper. All authors took part in critically reviewing and editing the manuscript.

## Conflict of Interest Statement

The authors declare that the research was conducted in the absence of any commercial or financial relationships that could be construed as a potential conflict of interest.
